# Supplementary material for: Are carbon emissions trading and green financial instruments synergistic? -Comprehensive quantitative research based on content analysis
Source: PLoS One. 2024 Mar 7;19(3):e0298601. doi: 10.1371/journal.pone.0298601 (PMC10919601; doi:10.1371/journal.pone.0298601)
Supplement: S1 Data — (ZIP) [file pone.0298601.s001.zip › Data/地级市Z_F工作报告.zip!/╣π╬≈/└┤▒÷╩╨/└┤╫╘╣1⁄2╓┌║┼í╛┬φ┐╦╩2╛▌═°í┐_2018.docx]

各位代表：

现在，我代表市人民政府，向大会作报告，请予审议，并请各位政协委员和其他同志提出意见。

2017年工作回顾

过去的一年，是我市发展史上极不平凡、极不容易的一年。这一年，在自治区党委、政府和市委的坚强领导下，我们全面学习贯彻党的十九大精神，迎难而上，积极应对，扭转了经济连续多年持续下滑的被动局面，呈现出止跌回稳、触底回升的良好势头。这一年，我们不等不靠、主动作为，突破了多年来产业发展一直想解决的瓶颈制约，实现了大工业区域电网、东线管道集中供热建成运行和创建国家级现代农业产业园的新突破。这一年，我们风雨兼程，砥砺前行，社会民生事业发展获国家、自治区多项表彰肯定，再获全国文明城市参评资格，成为广西首个义务教育均衡发展整市通过国家认定的设区市。这一年，是建市15周年，我们不忘初心，勤政为民，城市公共配套服务功能更趋完善，城乡人居环境明显改善，社会和谐稳定，市民幸福感稳步提升。

过去的一年，我们认真贯彻落实市委确定的“明大势、抓重点、守底线、用干部、善作为”总要求，按照“做大产业、做强工业、做精招商、做实项目”的工作重点，经过全市上下的奋力拼搏、克难攻坚，全年各项主要经济指标逐月企稳向好，我们正坚定地走在加快走出困境的路上。从一季度的全区排名末位，地区生产总值、规上工业增加值、固定资产投资、财政收入全都排在“黑榜”，到二季度部分指标排名升位进入“红榜”，再到三季度、四季度，全部指标跳出“黑榜”，甚至有些主要指标进入同比前三位，前后32次挤进了“红榜”，较好地完成或接近完成全年的各项目标任务。全年完成地区生产总值663.69亿元，同比增长7.4%，创近5年来最高增速，排全区第9位；固定资产投资432.16亿元，增长16.5%，在全区进步最快，排全区第3位；规上工业增加值增长5.9%，排全区第10位；财政收入48.25亿元，下降3.4%，排全区第11位；社会消费品零售总额180.29亿元，增长13.3%，排全区第1位；居民人均可支配收入19269元，实际增长7.9%；人民币存、贷款余额分别增长15.8%、15.2%；居民消费价格涨幅控制在1.5%以内。

过去的一年，一批国家级创建试点取得重大突破。国家售电侧改革试点和国家环境服务业试点成效显著，大工业区域电网竣工通电，东线集中供热管道正式供热，工业大用户天燃气直供管道正式开工，年产25万吨银海铝二期项目正式投产，铝产业“二次创业”翻开新篇章，实现了来宾市多年来实施“铝电一体”“热电联产”的夙愿。我市成为广西首个获批市级创建国家现代农业产业园的设区市，我市在全区现代特色农业示范区建设增点扩面提质升级动员部署会上作典型发言。荣获“全国未成年人思想道德建设工作先进城市”。金秀县荣获“全国森林旅游示范县”称号，列入商务部“电子商务进农村示范县”；兴宾区、象州县、武宣县、合山市荣获“全国青少年普法教育示范区”。

过去的一年，一批具有影响力的展会活动成功举办和一批具有标志性的重大决策举措启动实施。圆满承办2017中国甘蔗机械化博览会，并成功探索展会市场化运作模式，成功举办海外学人（企业家）回国创业及招商引资周和2017年广西（来宾）碳酸钙行业发展研讨会暨项目推介会，搭建开放合作平台，推介宣传来宾。“三区”管理体制、重大项目、招商引资、审批服务、国资国企、统计管理等体制机制改革不断深化。三江口港产城新区建设有序推进，“两场”下放我市管理工作获得自治区政府批准，市属平台公司加快进军产业领域，成立市碳酸钙管理办公室统筹推进碳酸钙产业发展，一批教育、文化、卫生、民政等社会公益性项目市场化运作扎实推进。驻柳原柳地“三大院”改造工作取得实质性进展。进一步聚集发展优势，形成推动发展的强大合力。

回顾过去一年，我们主要做了以下工作：

（一）持续加力稳增长，产业实力在突破短板中稳步提升。

工业实现恢复性增长。坚持做强工业，规上工业增加值增速在第二季度实现由负转正并不断攀升，全年完成规上工业总产值611.11亿元，增长17%，增速排全区第7位。加大服务企业力度，召开19次专题会，帮助企业协调解决60个问题，停产企业从年初的20家减少到6家，新培育规上企业9家。“一网两管”扎实推进，售电侧改革使园区企业用电成本每度下降0.1元左右，热电联产成效明显，实现供热产值5200万元，拉动用热企业产值约12亿元。新兴产业快速发展，实现产值82亿元，增长18.1%，其中已建成汽配企业11家，产值增长43.9%；碳酸钙加工企业42家，产值42.5亿元，增长30.8%，在建或筹建碳酸钙项目26个，总投资107.88亿元。银海铝二期、福美新材料二期、中科环保垃圾发电厂扩建、中沛电子等一批项目竣工投产。投入4亿元加快园区道路和一批污水处理工程等基础设施建设，全市工业园区引进项目39个，签约项目总投资97亿元；园区工业总产值495亿元，增长11%。创新驱动战略稳步实施，兴宾区红河红示范区获评全国创业创新基地，来宾高新园区获评自治区小微型企业创业创新示范基地，全市在孵企业78家，福美新材料公司等3家企业新认定为自治区企业技术中心，总数达到13家；新增高新技术企业8家，总数达到22家。获得授权专利270件，拥有有效发明专利247件，每万人口发明专利拥有量1.13件。深入实施“质量强市”战略，12个产品新获得广西名牌产品称号，为来宾历年之最。

现代农业加快发展。完善农田基础设施，桂中治旱一期、二期工程年内分别完成投资2.75亿元、7.85亿元，大藤峡移民安置工程年内完成投资31.1亿元，完成目标任务的3.11倍，基本扭转了这两项“国字号”工程进度偏慢的被动局面。水利基础设施投资24.47亿元，增长41.7%。新建成“双高”基地31.3万亩，累计达41.8万亩。新创建现代特色农业示范区92个，其中自治区级8个、市级10个、县级21个、乡级53个，实现全市66个乡镇示范区全覆盖。新增土地流转4.2万亩，累计达57.3万亩。全市农林牧渔业增加值增长4.6%。粮食产量81.55万吨，水果产量增长35.4%，肉类总产量增长1.8%，水产品产量增长4.9%，蚕茧产量增长5.6%。新增农民合作社1024家、家庭农场147家，分别完成目标任务的5.12倍和1.47倍。新增“三品一标”认证产品13个。发展富硒产业1.9万亩。农业机械总动力增加16万千瓦，农业机械化水平达58%，提高2.6个百分点。2017/2018年榨季开局良好，预计进厂原料蔗780万吨，增长17.3%，种蔗面积175.3万亩，新增11.7万亩，农民种蔗收入增长16.2%。农业招商力度加大，中粮集团等一批知名大企业逐步落户我市。

特色服务业持续旺盛。实现服务业增加值253.65亿元，增长11.5%，对经济的贡献率为57.9%，拉动经济增长4.3个百分点。全域旅游稳步发展，大瑶山少数民族生态旅游圈列入自治区层面重点打造，金秀入选国家全域旅游示范区创建单位，合山成功进入广西特色旅游名县创建行列，武宣百崖大峡谷通过自治区级生态旅游示范区评定，忻城薰衣草庄园获评自治区文化产业示范基地。以金秀为重点的民宿旅游加快发展。全市接待游客首次突破2000万人次，达2200万人次，增长21%；旅游总消费175亿元，增长31%。成功创建国家电信普遍服务试点，电信业务总量增长84.6%。邮政业务总量增长28.8%。新增限额以上入库企业23家。批发、零售业销售额分别增长9.1%、28%。来宾高新科技园、金秀县瑶都生态养生等2个服务业聚集区通过自治区认定。梧柳高速公路建成通车，贺巴高速公路（蒙山至象州段）开工建设，金秀县将结束不通高速的历史，象州经济发展将再添新翼；柳南高速公路改扩建工程加快推进；来宾至石龙等33个路网项目顺利推进。完成交通固定资产投资49亿元，增长18.9%；公路、水路客货运周转量分别增长9%、15.7%；港口货物吞吐量1152万吨，增长8.5%。实施“电商来宾”工程成效明显，全市建成县级电商服务中心3个，乡级服务站28个，村级服务网点391个，电子商务成为推动农村消费升级的新动力。房地产销售面积159.15万平方米，增长4.4%，平均价格增长7.8%。

供给侧改革推动产业质量提升。积极稳妥去产能，排查并逐步出清“僵尸企业”7家，退出煤矿产能72万吨。综合施策去库存，使用住房公积金16.3亿元促进住房消费，房地产去库存144.53万平方米，棚户区改造货币化安置1903套。防范风险去杠杆，直接融资占比达35%；实现小微企业贷款138亿元，增长25%；加强政府债务管理，向上级争取债券资金50.49亿元。多措并举降成本，落实新“28条”等系列降成本惠企政策，降低企业成本16.17亿元。协调发展补短板，完成基础设施建设投资152.96亿元；棚户区改造基本建成4754套；完成农村危房改造6213户。

（二）凝神聚力强后劲，项目建设在破解瓶颈中实干奋进。

创新投资工作机制，实行“前欠后还”、重大项目交办跟踪落实制度、项目前期工作总承包、精简优化审批流程等机制得到自治区充分肯定。发挥市重大办管理协调作用，建立项目“四级”协调机制，实行市、县四家班子及部门负责人联系项目制度。召开投资和项目专题协调会21次，解决项目建设问题229个。固定资产投资呈低开高走态势，同比回升33.9个百分点。其中，5000万元以上重大项目完成投资167.28亿元，增长57.5%；民间投资占全市投资额的49.2%。建立项目入库、储备机制，新增入库项目1273个，其中5000万元以上重大项目120个；储备项目2000多个，总投资1644亿元。实现110个项目开工建设，其中提前开工43个；有43个项目竣工投产，其中提前竣工24个。实行联动用地报批制度，全力落实项目建设用地，用地报批一次性审查通过率为83.3%，排全区第1位；获批新增建设用地1.8万多亩，是上年的10.3倍；盘活存量土地1.2万多亩；项目供地率73.5%，比自治区目标高13.5个百分点。创新投融资机制，加强筹融资工作，发挥好财政的统筹作用，推行市属平台公司体制机制改革，增强筹融资和自身发展能力，在有效防范和化解债务风险的同时，筹集资金12.32亿元投入项目建设。获得中央预算内投资11.2亿元；发行企业债券8亿元，新增社会融资100亿元。

（三）齐心协力增动能，招商引资在精准发力中风生水起。

坚持把招商引资作为做大产业、做强工业的关键一招。市、县（市、区）四家班子领导、园区及部门负责人带头外出“大招商、招大商”，坚持每半月发布一次市、县领导招商引资动态信息，营造比学赶超的浓厚氛围。创新政企组团联合招商、“1拖X”团队招商等多种招商模式，建立签约项目快速落地移交机制，我市投资环境满意度提高4位，排全区第3位，创近5年来最高。充分发挥“一网两管”优势，坚持推进以资源换产业，围绕支柱产业和新兴产业精准招商，瞄准重点企业、龙头企业招大引强，发挥驻京联络处的对外联络和招商推介作用，招商引资工作取得显著成效。年内签订招商引资正式协议100个，总投资412.81亿元，其中开竣工项目37个，投资总额123.5亿元。招商引资实际到位资金439亿元，增长11.1%，其中全口径实际利用外资1.6亿美元，增长12.7%。全年共引进78个投资亿元以上产业项目。

（四）汇聚合力夯基础，城乡发展在区域协调中绽放活力。

坚持完善基础，提升品质，促进城市更靓、乡村更美、环境更优。市主城区建成区面积46平方公里，预计城镇化率达44.5%，提高2.3个百分点。着力破解城市建设征地搬迁安置难题，集中力量开展征地搬迁“百日攻坚大会战”“征地搬迁服务月”和“冬季征地搬迁安置大会战”等活动，完成征地1.42万亩，同比增加3016亩；开工建设9条断头路，解决了多年因征地搬迁原因无法打通的道路瓶颈，建设来宾北站出入口通道雨棚便民工程、增加市区机动车停车位等便民措施得到市民称赞；实施滨江北路西段、江滨路及配套绿化项目，推进红水河防洪堤等沿江项目建设，解决我市市区“靠水不亲水”的现状，停工多年的世行项目草鞋沟河道整治恢复开工，桂中水城维林渠启动建设；盘活停建楼盘30万平方米，依法处置78宗约5550亩闲置土地；推进大桥路等12条老城区道路改造。城北新区服务功能提升配套项目完成投资13.05亿元，一批学校、道路、社区卫生服务中心相继竣工并投入使用；完成26个社区建设大会战项目。驻柳管理服务水平得到提高。继续推进来宾市国家新型城镇化综合改革试点等工作，新增农业转移人口市民化6万人，农民工等新市民首次购房面积32.3万平方米。开展“美丽来宾•宜居城市”创建活动，完成植树造林26.78万亩，森林覆盖率52.12%，金秀县纳入国家重点生态功能区；市城区种植木棉树2万株，改造提升凤凰山、老梅沟、蓬莱洲森林公园，市城区绿化覆盖率30.1%。加强与广西林业集团合作，提升我市林业产业发展水平。全面推行“河长制”，全市1226条河流均设立了河长，明确市县乡村四级河长2946名，境内主要河流水质全部达标。二氧化硫、氮氧化物排放量分别下降14.5%、12.2%，预计万元生产总值能耗下降3.66%。开展城市环境整治百日大行动，拆除违章建筑12.7万平方米，清理建筑垃圾约5821吨；整治占道经营及跨栏经营2.73万户（次），清理流动夜市摊点1.7万家（次），市容市貌得到明显改善。完成20个镇级污水处理设施项目、58个农村垃圾专项治理攻坚项目建设，市城区污水处理率在89%以上。开展“美丽来宾·宜居乡村”建设活动，创建市级“宜居乡村”综合示范村36个，完成改厨8.39万户，改厕8.37万户，改圈764户。县域经济实力进一步提升。金秀县荣获广西科学发展进步县，地区生产总值增长10.1%；兴宾区增长6.6%；象州县增长10.3%；武宣县增长7.7%；忻城县增长5.9%；合山市增长5.4%。河南工业园区、来华管理区（来宾高新区）规模以上工业增加值分别增长3.1%、6.8%。

（五）群策群力齐帮扶，脱贫攻坚在披荆斩棘中扎实推进。

大力开展精准扶贫精准脱贫，投入各级财政专项扶贫资金7.55亿元，整合各类财政涉农资金11.67亿元用于产业扶贫、基础设施建设、生态保护、村集体经济、易地扶贫搬迁等项目，其中投入7916万元，发展各类特色种养产业，惠及贫困群众3.72万人；修建贫困村屯级道路64条112.7公里、人饮工程130处。年内累计完成2016年、2017年易地扶贫搬迁项目投资17.3亿元，建设集中安置点31个，累计搬迁安置2.75万人。年内减少贫困人口4.56万人，脱贫摘帽51个贫困村，圆满完成自治区下达的年度目标任务。合山市扶贫经验得到国家领导人肯定性批示。

（六）竭尽全力保民生，和谐社会在固本强基中普惠共享。

坚持以人民为中心的发展思想，一大批惠民举措落地实施，群众获得感明显增强。全市投入民生资金146.2亿元，占支出总额的81.3%。教育事业加快发展。学前三年毛入园率、义务教育巩固率、高中阶段毛入学率超额达标并稳步提升。一批新建学校建成招生，消除城区义务教育学校“大班额”攻坚成效明显，全面消除“超大班额”，提前一年完成自治区目标任务，得到市民群众的支持肯定。六个县（市、区）义务教育均衡发展顺利通过国家评估认定，率先在广西实现“国检率”100%，市县教育投入达98.5亿元，提前三年完成自治区目标任务。加快推进高中规划建设，启动柳州地区民族高中迁建项目，来宾一中迁建项目开工建设，来宾实验高中迁建项目基本完工。学前教育扩容提质，全市新建公办幼儿园20所，启动来宾市实验幼教集团项目建设。中等职业学校在校生达2.39万人，全面完成来宾职业技术学院组建。广西科技师范学院不断发展，“厅市共建”工作启动实施，全日制在校生达1.28万人，增长26.8%。公共文体服务水平不断提高。获评“第七轮自治区文明城市”，市气象局、市地税局、来宾供电局获“全国文明单位”称号。建成新市图书馆、档案馆、群众艺术馆，建设59个村级公共服务中心，实施53个行政村有线广播电视网络覆盖工程，文化惠民演出1200场。我市荣获“全国群众体育先进单位”称号，获列广西全民健身和全民健康深度融合试点市。成功承办2017年广西“壮族三月三·民族体育炫”来宾市主会场系列活动和广西群众高水平篮球精英赛，成功举办来宾市第一届少数民族传统体育运动会。组织举办各种体育赛事1000多场，进一步丰富群众生活。我市运动员在各级各类比赛中，荣获全国金牌19枚、银牌16枚、铜牌15枚，为我市争得了荣誉。地方志、残联、老龄等工作扎实有效开展。卫生与计生工作扎实推进。有序推进分级诊疗，构建多种形式的医疗联合体，探索引进社会资本和技术力量与公立医院合作办医新模式；妇幼保健工作名列全区前茅；疾病预防控制工作成效显著；基层医疗卫生服务能力建设得到新提升。全面两孩政策平稳实施，人口自然增长率7.35‰，全面完成人口计生各项指标任务。社会保障再上新台阶。全市城镇新增就业1.2万人，农村劳动力转移就业3.3万人次，城镇登记失业率2.6%，低于控制数1.5个百分点。累计支出救助资金3.39亿元，救助困难群众159.5万人次。筹措资金7472万元，实施滨江小学、汽车客运中心自动售票系统等市级10项为民办实事工程。完成农村电网改造投资4637万元。市外侨办获“全国侨办系统先进集体”。市妇联荣获“全国维护妇女儿童权益先进集体”，工会、团委等群团组织作用进一步增强。社会公共安全管理成效明显。荣获“建设平安广西活动先进市”“广西无传销城市”称号，群众安全感和满意度保持全区前列。实施科技强警，智慧警务支撑作用明显，刑事案件下降16.5%，治安案件下降15.5%，市区“两抢”案件警情下降29.68%，零发案天数达275天。“三大纠纷”案件调结率91.9%。全市信访批次、人次分别下降40.9%、40.3%。实现村（社区）法律顾问全覆盖，办理法律援助案件1424件、诉讼案件1240件。安全生产持续稳定，事故起数、死亡人数下降20%和27%。扎实推进自治区食品安全示范城市创建。基层应急管理体系不断完善，开展防空、地震、消防、反恐、危化品、金融等应急演练，妥善处置各类突发事件。

（七）不遗余力优环境，行政服务在创新机制中提速增效。

改革创新积极推进，上级在我市实施的16项“先行先试”改革试点进展顺利，财税体制、公立医院、不动产登记、土地确权登记等重大改革持续深化。深化推进乡镇“四所合一”改革，减少办事环节115个；实现工商登记“35证合一”；文化、教育、司法、供销、国有林场等其他领域改革有效推进。深入推动行政审批制度改革和“放管服”改革。以开展“解放思想，改进服务，提高效率”专项整治活动为契机，深刻剖析典型个案，吸取教训，对39个市直部门的552项行政许可和公共服务事项办理流程进行优化再造，实行“审批窗口处级领导坐班制”，取消、减少申报材料214项，“马上办”事项93项，1天内办结事项113项，承诺办理时限比法定办结时限整体提速89.9%。全市新增实有企业3583家，增长24.6%，新增个体工商户1.39万户，增长25.1%。法治政府建设成效明显，自觉接受市人大、政协监督，办理人大代表建议73件，政协提案104件，办结率均达100%。全力落实去年人大通过的《政府工作报告》各项目标任务，采取每季度召开一次督查推进会、市政府领导带队督查等措施，确保各项工作任务落到实处。同时，提请市人大委托第三方对市政府落实去年《政府工作报告》的各项内容进行评估监督。加强市政府党组建设和理论中心组学习，认真履行全面从严治党主体责任，先后召开7次政府系统落实全面从严治党主体责任典型案例剖析会，锲而不舍落实中央八项规定精神，坚决纠正“四风”，党风廉政建设和反腐败工作、意识形态责任制工作扎实推进。国防动员和后备力量建设、机关事务、保密等工作取得新进展。

各位代表，过去的一年，困难多、任务重、挑战大，我们沉着应对、合力攻坚，稳住了经济，提升了信心，有目标、有奋斗、有艰辛，不容易、不简单、不平凡。这是自治区党委、政府和市委坚强领导的结果，是市人大、市政协监督支持的结果，是社会各界和有关方面热情参与的结果，更是全市上下奋力拼搏的结果。我们要为全市人民点赞，为人大代表、政协委员点赞，为驻市的中直、区直部门和人民解放军、武警官兵，为各民主党派、无党派人士、工商联、各人民团体和各界人士，为所有关心支持来宾建设发展的港澳台同胞、海外侨胞及国际友人点赞。在此，我代表市人民政府，向大家表示衷心的感谢并致以崇高的敬意！

需要说明的是，受经济下行压力、历史欠账、动能转换周期等因素影响，地区生产总值、工业增加值等指标增速略低于预期，财政收入指标离预期目标尚有较大差距。但是我们清醒地看到，经济社会发展还面临不少困难和挑战，全市最大的实际是发展不平衡不充分，最突出的短板是基础设施薄弱，最根本的症结是发展基础不牢，表现在：新旧动能转换不快，新的增长动力不足；工业依然艰难地处在走出困境的路上；现代农业产业化仍然滞后，产业、产品层次不高；民间投资不足，创新能力不强，新经济、新业态发展不快；财政收入增长乏力，债务压力大；脱贫攻坚任务十分艰巨；民生事业、社会稳定等热点难点问题还没有得到根本解决；少数干部不担当、不作为、慢作为，机关效能有待提高等等。对此，我们一定正视矛盾，强化措施，解决问题，决不辜负全市人民的重托。

2018年目标任务

2018年是全面贯彻落实党的十九大精神的开局之年，是改革开放40周年和自治区成立60周年。中国特色社会主义进入了新时代，新时代开启新征程，新时代呼唤新作为。在新的历史方位下，我们要以永不懈怠的精神状态和一往无前的奋斗姿态，攻坚克难，继续奋进，推动来宾经济尽快走出困境和社会事业再上新台阶。

新时代我们重任在肩。党的十九大开启了全面建设社会主义现代化国家新征程。前进的号角已经吹响，进军的旗帜已经高擎，我们必须跟随旗帜奋勇前进。从国内外、区内外形势来看，世界经济正复苏增长，我国坚持稳中求进工作总基调，贯彻落实新发展理念，按照高质量发展的要求，以供给侧结构性改革为主线，建设现代化经济体系，实施乡村振兴战略和区域协调发展战略，推动经济发展质量变革、效率变革、动力变革，经济增长的前景更加广阔，发展潜力得到充分释放。自治区深入贯彻落实习近平总书记赋予广西“三大定位”新使命和“五个扎实”新要求，着力打造由重庆经广西出海连通新加坡的“渝桂新”南向通道，构建西部地区南北走向大动脉，持续营造“三大生态”，加快实现“两个建成”，将制定出台一系列抓重点、补短板、强弱项的政策措施。这些将为我市加快发展创造良好的环境和有利条件。我们要紧跟新时代发展步伐，坚持把发展作为第一要务、把做大产业作为兴市之要、把创造美好生活作为不懈追求，乘势而上，苦干实干，蹄疾步稳踏上赶超跨越的新征程。

新时代我们充满信心。从我市经济发展的走势来看，总体判断是：今年的发展形势要比去年好。大工业区域电网正式运行，“铝电一体”“热电联产”成为加快发展和招商引资的强劲优势，银海铝二期、福美生态材料二期等一批重点产业项目竣工投产，将新增工业产值90亿元以上，实现利税2亿元左右；桂中治旱工程、大藤峡库区移民安置、三江口港产城新区基础设施建设等一批重大项目加快推进，将新增固定资产投资48亿元；铝精深加工产业园、碳酸钙百亿元产业、建筑新材料产业、国家现代农业产业园、现代农机产业园等新动能加快培育，成为稳增长的新支柱；广铁公司等现有企业加快改革步伐，将释放出新的活力；一批招商项目相继落地开竣工并发挥效益，成为推动经济增长的强大动力。支持经济继续保持稳中向好的积极因素在累积增多，我们有可能、有条件、有信心实现经济社会平稳较快发展。

今年政府工作的总体要求和主要预期目标是：全面贯彻落实党的十九大精神和中央、自治区经济工作会精神，以习近平新时代中国特色社会主义思想为指导，结合来宾实际，按照市委“明大势、抓重点、补短板、强弱项、守底线、用干部、善作为”的总体要求，坚持稳中求进工作总基调，坚持新发展理念，按照高质量发展的要求，以供给侧结构性改革为主线，集中力量做强现代特色农业、资源深度开发、生态旅游发展“三块长板”，下大力气补齐脱贫攻坚、基础设施、公共服务“三块短板”，统筹推进稳增长、促改革、调结构、惠民生、防风险各项工作，为推动来宾经济扎实走出困境、社会更加和谐发展、人民群众逐步过上美好生活打下坚实基础。实现地区生产总值增长7%以上，固定资产投资增长12%，规模以上工业增加值增长7%左右，财政收入增长3%，社会消费品零售总额增长10%左右，进出口总额增长12%，城镇居民人均可支配收入增长6.5%左右，农村居民可支配收入增长9.5%左右，居民消费价格总水平涨幅控制在3%以内，城镇登记失业率控制在4.1%以内，常住人口城镇化率46%，人口自然增长率控制在10‰以内。

围绕我市“十三五”规划和实现全面建成小康社会目标任务，我们要认真贯彻落实市委工作部署和要求，切实践行新发展理念，明确目标、突出重点、主题不变、镜头不换，保持定力持续推进“做大产业、做强工业、做精招商、做实项目”，着力打造“四做”升级版。做大产业就是要加快建设现代产业体系，实施质量引领、创新驱动战略，坚持做优存量，做大增量，加快产业结构优化调整，推动传统产业转型升级，新兴产业培育壮大，今后3年地区生产总值年均增长速度要高于全区平均水平。做强工业就是要着力构建以优势传统产业为基础、新兴产业为引擎的现代工业发展体系，充分发挥“一网两管”优势，推动新旧动能转换，促进工业经济提档升级、做大做强，今后3年规模以上工业增加值年均增长速度处于广西中上水平。做精招商就是要充分利用资源和产业优势，招大商、引强商，精准跟踪服务，确保引得进、落地快、发展好，不断扩大经济规模。今后3年招商引资到位资金实现年均增长15%以上。做实项目就是要紧盯项目谋划、前期、开工、建设、竣工等环节，实施产业、基础设施、社会公益“三个100”工程，加快推进项目建设，不断增强发展后劲，今后3年固定资产投资年均增长速度处于广西上游水平。

围绕上述目标任务，我们要着重抓好以下九个方面工作：

（一）围绕创新升级，挺起工业经济的坚强脊梁。

实施主攻工业三年行动计划。把工业发展放在全局工作的首位，做大工业经济。2018年重抓巩固企稳回升，突出 “一网两管”夯实产业竞争基础，突出设施建设夯实园区承载基础，突出工业招商夯实项目储备基础，突出项目建设夯实持续发展基础。到年底工业经济总体上要摆脱困境，实现工业总产值700亿元，增长13.3%。2019年重抓发挥优势做大总量，重点发展电力、铝及精深加工、制糖、碳酸钙、茧丝绸等支柱和优势产业，逐步形成产业集群，工业经济增速达到全区中等水平，实现工业总产值850亿元，增长21.4%。2020年重抓冲刺千亿元目标，实施工业规模、创新能力、产业结构、智能制造、绿色发展等五大攻坚计划，工业经济增速进入全区中上水平，力争工业总产值突破1000亿元，增长17.6%以上。加强工业布局梯队推进，市河南工业园作为龙头，“十三五”末工业总产值要突破300亿元，形成第一梯队；武宣县、象州县、兴宾区、来华管理区（来宾高新区）作为中坚力量，形成第二梯队，“十三五”末工业总产值要分别突破100亿元，总计达550亿元左右；忻城县、金秀县、合山市作为后发力量，形成第三梯队，“十三五”末工业总产值要分别突破50亿元，总计达150亿元左右。三江口港产城新区在加大基础设施建设的同时，聚焦产业，做大工业，“十三五”末工业总产值达50亿元。

壮大产业发展实力。坚持资源换产业、存量促增量的原则，延伸资源开发产业链，推动实体经济发展。着力抓存量升级。全年完成工业投资120亿元以上，稳步推进冶炼、制糖、能源、铝及精深加工等传统优势产业改造升级，重点加快一批龙头企业、重大项目建成投产。实现西线管道集中供热建成运行。加快企业改制重组，重点推进广铁公司、合山煤业公司重组工作，推动恢复生产；支持实力强的企业集团兼并重组小糖厂，形成4家大型企业集团，年产糖量100万吨以上。着力抓增量扩大。培育壮大中小微企业，新增规上企业12家以上。积极发展碳酸钙、汽车（机械）零配件制造、茧丝绸及服装、长寿健康养生、生物及民族医药、新材料等新兴产业，新兴产业产值增长14 %。

完善提升工业园区。加大资金投入，重点建设福美新材料、汽车电子、碳酸钙、农机装备、茧丝绸等特色园区，改善基础设施，提高承载能力。加快园区规划修编，保障工业项目用地需求。加大三江口港产城新区招商引资力度，开工建设莆田大桥、正龙大桥、黔江大桥、高安至柳武高速黄茆出口公路“3桥1路”等基础设施，完成投资2亿元。完成园区基础设施投资5亿元以上，园区工业总产值增长11%。

增强创新创业能力。深入实施创新驱动战略，突出来华管理区（来宾高新区）在高新产业发展方面的领军作用。强化企业创新主体地位，支持我市科技企业孵化器和众创空间的建设和发展，建设和培育恒达电机等3个工程技术研究中心。完成重大科技成果转化10项以上；新增高新技术企业5家以上；发明专利申请量和拥有量分别增长10 %和5%以上。积极开展质量品牌行动，大力实施标准化战略，弘扬企业家精神和“工匠精神”，支持我市企业争创广西名牌产品6个，组织制（修）订广西地方标准5项。

优化企业发展环境。发挥好政企互动平台作用，强化企业跟踪服务。落实好自治区“41条”“28条”和我市“38条”降成本惠企政策，切实降低企业运行成本；加强政银企对接，解决企业融资难题；开展好“春风行动”、校企合作、常态化招聘等活动，满足企业用工需求，为企业提供用工5000人以上。

（二）围绕乡村振兴，打造现代特色农业新引擎。

着力实施乡村振兴战略。按照产业兴旺、生态宜居、乡风文明、治理有效、生活富裕的总要求，根据实施乡村振兴战略明确的目标任务，建立健全城乡融合发展的体制机制，加快推进农业和农村现代化，让农民有更多的获得感和幸福感。

着力推进现代特色农业示范区建设。以创建来宾市国家现代农业产业园为重点，扎实开展现代特色农业示范区建设增点扩面提质升级行动，完成现代农业产业园路网等基础设施建设，加快引进一批深加工、仓储、物流等项目，推动一二三产业融合发展，将现代特色农业打造成为富民兴市的新引擎。

着力健全现代农业产业体系。深化农业供给侧结构性改革，实现农林牧渔业增加值增长4%。落实好粮食安全行政首长责任制，稳定粮食产量。加快“双高”糖料蔗基地建设，新增“双高” 基地55万亩，总面积达95万亩，实现进厂原料蔗900万吨以上；加快建设优质肉牛、肉羊、水奶牛、生猪、水产等特色良繁养殖基地，扩大规模化生态养殖，实现肉类总产量增长3.6%，水产品产量增长5.9%；着力推进精品水果、蔬菜种植，打造桂中特色果园、绿色菜篮子基地，实现水果产量增加9万吨，蔬菜产量增加6万吨；实施规模化、标准化桑园建设，实现鲜茧产量7.7万吨；推动林业立体化发展，林下经济综合产值超30亿元。

着力健全现代农业生产体系。强化农业基础设施建设，完成桂中治旱工程一期投资8000万元、二期投资9亿元的建设任务。扎实推进大藤峡移民安置工程建设，完成投资10亿元以上。重点推进农田小水利、病险水库除险加固等水利工程建设，完成投资15亿元。推动农业科技创新及成果转化，加快甘蔗收种机械化步伐，农机总动力达220万千瓦以上，农作物耕种收综合机械化水平达60%。推进农业绿色发展，扩大标准化生产规模，农产品质量例行监测平均合格率达97%以上。加快推进一批农产品品牌建设，打造富有竞争力的农产品品牌影响力。

着力健全现代农业经营体系。完善农村土地制度，全面完成农村土地确权登记颁证工作，稳步推进农村集体产权制度改革。扩大新型经营主体规模，完成培育市级以上示范社、家庭农场30家以上。加强农业生产社会化服务，建立完善服务体系。加强农村人才队伍建设，培育新型农民，发挥“土专家”作用，引导返乡农民工和大学生到农村创业，推动农业农村新发展。

着力推进美丽来宾乡村建设。深入开展“宜居乡村”活动，启动农村人居环境整治三年行动。创建市级40个“宜居乡村”综合示范村，完成改厨、改厕、改圈重点任务，大力推进“厕所革命”，实行公共厕所“所长制”，确保农村厕所实现无害化。谋划启动“美丽来宾·幸福乡村”活动，打造美丽来宾乡村建设升级版。

（三）围绕特色优势，打造服务业发展新高地。

推动重点领域突破。大力发展特色服务业，完成服务业增加值增长9%。做大商贸物流业。完善方盛广场、大洋购物广场、迎宾广场周边商业设施，新增培育商贸限上企业10家以上，着力满足消费者多样化需求；积极培育新兴业态，引导大型实体零售与网络零售融合发展，打造线上线下融合发展的新型市场主体。加大对传统运输业的扶持力度，积极探索建立新型物流体系，促进运输业转型升级，公路、水路客货运周转量分别增长10%、18%，港口货物吞吐量增长10%。做精生态旅游业，推动合山市创建广西特色旅游名县、金秀县创建国家全域旅游示范区和武宣县、忻城县创建全区全域旅游示范县，积极推进民宿旅游发展，深化区域旅游合作，打造桂中生态旅游圈品牌，完成1个国家5A级、2个国家4A级景区申报评审和2个3A级景区评定，新增星级乡村旅游区或农家乐8家以上，全年接待游客增长15%，旅游总消费增长30%。加强文化与旅游深度融合，深入挖掘文化资源，发展特色文化产业，提升文化产业市场化发展水平。

推动平台载体建设。推进高新区科技园、金秀生态养生等服务业聚集区扩容提质，引导一批竞争力强的龙头企业入驻，提高聚集区集散能力。加快西江现代仓储物流园、来宾汽贸园、武宣东盟农副产品批发市场等大型商贸物流项目建设，支持已建成的专业市场和商贸城扩大招商，培育新的消费平台。加快电子商务进农村步伐，整合乡镇流通网络资源，搭建农村消费新平台，城乡电子商务服务网点达450个以上，促进电子交易额稳步提升。支持连锁化、品牌化企业优化网点布局，打造便民生活服务圈。

推动其他服务业发展。引导批发、零售、餐饮、住宿业转型发展，增强消费拉动能力。完善提升金融服务业，争取更多银行、证券、保险公司等金融机构在我市设立分支机构。促进房地产业健康发展，完成商品房销售面积160万平方米。加强物业行业监管。积极发展设计咨询、资产评估等中介服务业。实施好养老示范园区建设工程，加快养生养老服务业发展，打响长寿品牌。推进两化融合发展，加快来宾市电信普遍服务试点建设。

（四）围绕挖掘潜力，推动发展活力持续释放。

强化项目支撑。坚持扩大有效投资，实施重大项目建设“三个100”工程，即产业项目投资100亿元以上、基础设施项目投资超过100亿元、实施100个以上社会公益项目。全年重大项目完成投资243亿元以上。全年实现120个项目开工；加快153个续建项目建设；力争45个项目竣工；精心谋划储备项目，争取更多项目列入国家和自治区层面推进，投资超亿元滚动储备项目共136项，总投资923.7亿元。加快交通项目建设，完成投资34亿元以上。加快柳州到来宾城市轨道交通项目前期工作，开展线网规划编制。力争完成柳南高速（来宾段）改扩建工程，推动柳州经合山至南宁高速公路（来宾段）进入施工阶段，完成600公里等级路建设目标，把农村公路建好管好护好运营好。加快推进兴宾莆田作业区、武宣樟村作业区等前期工作，推进武宣龙从作业区、象州钓鱼公作业区进港公路等港口配套项目建设。

强化改革带动。以改革增动力、提效率。深化供给侧结构性改革，重点在“破”、“立”、“降”上下功夫，大力破除无效供给，培育新动能，降低实体经济成本。持续推进“三去一降一补”，继续推进“僵尸企业”清理工作，保持商品住房消化周期在12个月以内，直接融资比例达25％以上，降低企业成本6亿元以上。深化重点领域改革，推进和完善“三区”管理体制改革，推动市与城区协调发展，激发园区发展内生动力。加大市属国有企业改革力度，推动国有企业在“做大产业、做强工业、做精招商、做实项目”中发挥更大作用，提高企业盈利能力。强化国有资产监管体系建设，确保国有资产保值增值，建立健全激发企业活力的体制机制。着力抓好医药卫生体制、养老服务、农村金融、事业单位、乡镇行政管理体制等领域改革，增强经济发展活力。

强化开放引领。坚持精准招商，继续引入招商引资竞争机制，积极探索外包招商、委托招商、以商招商、政府购买服务等新型招商模式。加大现代农业及农产品深加工、新兴产业、现代服务业、现代农机、碳酸钙、铝精深加工等重点产业精准招商力度；引进并依托有实力的大国企，开展以商招商；锁定珠三角、长三角、京津冀等经济发达地区和中国500强龙头企业，做实招大引强；推进县域重点产业与重点园区融合发展，组织特色招商。充分发挥驻京联络处的对外交流和联络作用，设立驻广州招商专门联络机构和聘请招商法律顾问。力争招商引资到位资金500亿元，增长15%以上，其中全口径实际利用外资1.6亿美元；实现100个以上投资超亿元产业项目落地开工，其中市本级（含河南工业园、来华管理区）完成40个以上，六个县（市、区）各完成10个以上。

强化要素保障。加强市属平台公司建设，增强筹融资能力，在有效化解债务风险的同时筹集资金投入项目建设。加大金融对实体经济和项目建设的支持力度，力争全社会融资总量达110亿元，新增贷款55亿元以上；采取政府和社会资本合作（PPP）、总承包（EPC）等模式，引入社会资本参与各类项目建设。保障项目建设用地，清理盘活存量建设用地，最大限度满足重点项目、重点企业建设用地，争取获得建设用地指标1万亩以上。加强税源培植，深挖税收潜力，提升财政保障能力；强化政府性债务风险预警，稳妥化解风险。深入实施“万才返乡·共建小康”计划和人才小高地建设提升工程，加快推进本土人才培训和队伍建设。

（五）围绕共建共享，推动城乡互动协调发展。

促进空间布局优化升级。推动城市集约发展，全面完成市总体规划修编和文化体育设施等6项专项规划编制。推进凤凰、良江、迁江片区控制性详细规划编制；推进城市道路综合交通规划、城市绿道系统总体规划、整体城市设计方案等编制。

促进新型城镇化建设升级。加快来宾市和象州、武宣县等国家级有关城镇改革试点建设。坚持“房子是用来住的，不是用来炒的”，有序控制房地产开发，积极引入一线房地产企业，提升我市房地产品质；实施“一企一策”，加快盘活处置市城区房地产停建项目；推进滨河北片区、来宾电厂生活区等棚户区改造项目。加快培育一批特色小镇。

促进城乡环境宜居升级。全面推进“美丽来宾•宜居城市”创建活动，继续开展城北新区完善提升工作，完成投资15亿元；加大征地搬迁安置工作力度，推进旧城区改造工作，开工建设连接旧城区的滨江北路东段，打通维林大道延长线、中南路延长线、桂中大道延长线等9条城区“断头路”；推进江滨路、向阳西路等5个地下综合管廊项目建设；开工建设来宾园博园。推进城乡污水垃圾处理设施建设，确保市城区污水处理率达90%以上，县城污水处理率达85%以上，城区污泥无害化处置率达75%以上，完成年度镇级污水处理设施建设任务。做好城市绿化美化工作，实现市城区绿化覆盖率达30.6%；重点推进草鞋沟、维林渠等项目，基本完成桂中水城建设；加快推进合山洛灵、忻城乐滩水库、兴宾三利湖国家湿地公园建设。完成造林面积20万亩，新增种植木棉树1万棵，森林覆盖率达52.4% ；实施蓝天碧水工程，打好“蓝天保卫战”，全面落实“河长制”“湖长制”责任，完成自治区下达改善空气质量、万元生产总值能耗、节能减排目标任务，进一步提高城乡宜居环境。

促进城市管理转型升级。进一步理顺市与城区市政管理体制，推行完善“街长制”，建立城市管理长效机制，切实解决交通堵塞、环境污染、街道脏乱、占道经营等问题。充分发挥驻柳管理处的服务和协调作用，有序推进驻柳原柳地“三大院”的改造重建。继续推进智慧城市建设，提高城市管理信息化水平。

（六）围绕脱贫摘帽，推动脱贫攻坚加速发力。

突出产业扶贫主力军作用。大力培育发展特色优势产业，每个贫困县要培育2至5个对贫困村贫困户覆盖面广的特色产业，每个贫困村要发展1至3个特色产业且覆盖60%以上贫困户。充分发挥种养大户、家庭农场、农民合作社等新型农业经营主体带动作用，依托特色产业引进培育一批龙头企业，引导贫困户积极参与，确保贫困户都有合作社或龙头企业带动。大力推进旅游扶贫，打造旅游扶贫升级版。加大金融扶贫力度，撬动金融资本和社会资本参与扶贫。大力发展贫困村集体经济，确保年内预脱贫摘帽的贫困村集体经济收入达到3万元以上。全市年内完成55个贫困村摘帽、6万贫困人口脱贫，武宣县实现脱贫摘帽。

突出基础设施建设带动作用。结合“美丽来宾•宜居乡村”建设活动，整合资源，加大投入，不断完善贫困村道路、饮水、住房、电力、通讯等基础设施，尽快补齐贫困地区基础设施“短板”。用足用活国家和自治区政策，切实抓好中央预算内资金和易地扶贫搬迁工作，根本扭转我市这两项“硬任务”在全区排名靠后的被动局面，完善后续扶持方案，做到实施一个项目，安置一方群众。落实好集中安置点“八包”责任制，加快推进自治区下达的2018年项目建设，全面完成2017年度建设任务扫尾工作。

突出社会扶贫助推作用。大力推进教育扶贫和就业扶持工作。深入实施“雨露计划”和学生资助行动，做到应补尽补。不断加强职业技能培训，提高贫困群众的谋生技能，引导贫困群众就地就近就业、返乡创业，实现增收脱贫。进一步强化“一帮一联”责任，加大领导挂点、部门包村、干部帮户力度，确保帮扶工作真正落到点上、扶到根上。鼓励支持民营企业、社会组织、个人参与扶贫开发。大力推进社会救助工作，通过全面落实特困人员、“低保”、医疗救助等政策，发挥政策保障作用兜底一批。利用好粤桂扶贫协作关系，加强与广东茂名市的信宜市、茂南区的对口扶贫，助推我市忻城县、金秀县深度贫困地区精准脱贫。

（七）围绕扩量提质，推动县域经济平衡充分发展。

全力做大产业。实施好县域经济发展三年行动计划，通过做大产业，带动县域经济更平衡更充分发展。兴宾区要深入发展现代农业、现代服务业、碳酸钙等产业，实现生产总值增长7.5%以上。象州县要大力发展新能源、汽配加工、农产品深加工等产业，实现生产总值增长7%。武宣县要抓好碳酸钙、港口物流、特色农业等产业，实现生产总值增长7%。忻城县要加快推进石材、茧丝绸、旅游等产业，实现生产总值增长6.5%。金秀县要大力发展生物医药、农产品深加工等特色产业，打造品牌旅游业，实现生产总值增长7.5%。合山市要强力推进“热电一体”、碳酸钙等产业，实现生产总值增长6.5%。来华管理区（来宾高新区）和河南工业园区要大力发展高新技术产业、先进制造业、铝精深加工等产业，不断推动产业壮大发展，分别实现财政收入增长15%、10%。

全力做强工业。兴宾区要加快小平阳碳酸钙产业园、莆田工业园区以及三江口新区高安、正龙片区的开发建设，推动汇宾钙、日昌升建筑新型骨料、裕达装配式建筑项目建成投产，实现工业增加值增长12.5%。象州县要加速推进石龙片区、马坪片区建设，打造桂中森林工业城，工业增加值增长7.5%。武宣县要打造矿产品精深加工基地、碳酸钙产业基地和区域物流中心，工业增加值增长7.5%。忻城县要做大做强石材、茧丝绸等主要产业，工业增加值增长9%。金秀县要重点推进桐木、头排工业园区建设，工业增加值增长12.5%。合山市要巩固火电、热电产业发展，大力推进合忻碳酸钙产业园区建设，工业增加值增长12.5%。来华管理区（来宾高新区）和河南工业园区要担当全市工业发展的主力军，工业增加值分别增长7 %和6%。

全力做精招商。以大招商、精招商，招大商、招强商论英雄，推进招商引资工作向更精准，更具针对性、可操作性发展，提高招商项目的签约率、开工率、竣工率。通过招商引资培植壮大税源，进一步壮大县域经济规模。兴宾区实现到位资金增长12.8%，象州县增长12.7%，武宣县增长13.3%，忻城县增长12.5%，金秀县增长25%，合山市增长15.2%，来华管理区（来宾高新区）增长18.6%，河南工业园区增长17.7%。

全力做实项目。坚持以项目建设为核心抓手，增强县域经济动能。实现固定资产投资兴宾区增长10%，象州县增长14%，武宣县增长13%，忻城县增长13%，金秀县增长14%，合山市增长8%，来华管理区（来宾高新区）增长15%，河南工业园区增长17%。

（八）围绕安康和谐，推动社会民生稳步改善。

强化社会保障体系。增进民生福祉是发展的根本目的，实施好城乡居民收入倍增计划，不断满足人民日益增长的美好生活需要。实现城镇新增就业1万人，转移农村劳动力2.7万人。稳步推进机关事业单位养老保险制度改革及异地就医、大病保险改革。加强劳动保障监察执法，重点打击拖欠农民工薪酬等行为。全面提升防灾减灾救灾能力，抓好城乡低保扩面提标，做好社会救助托底保障工作。实施农村危房改造4074户，开工建设城市棚户区改造4054套，解决困难群体的住房问题。进一步深化养老服务业综合改革实验区建设，完善市民政福利园功能布局；加快推进社区养老服务机构建设；兴宾区、来华管理区（来宾高新区）、市河南工业园区各建1所社区养老服务示范机构，推进养老机构公建民营改革。促进社会福利和慈善事业发展，全年安排困难残疾人生活补贴和重度残疾人护理补贴120万元以上。完成社区服务场所建设27个。新实施10项为民办实事工程。

完善公共服务体系。优先发展教育事业。落实立德树人根本任务，加大未成年人思想道德建设，加强师德师风建设，培养高素质教师队伍。在消除“超大班额”的基础上，全面完成消除城区“大班额”攻坚任务，开工建设市九中、十一中、十二中等初中以及富华、平西、古三等小学。办好学前教育，切实解决市城区“入园难”问题，开工建设来宾市第二、第三实验幼儿园等优质公办幼儿园，新建翠屏等高标准优质民办幼儿园。统筹推进县域城乡义务教育一体化改革发展，促进义务教育由县域基本均衡向市域优质均衡发展。加快推进柳州地区民族高中、来宾一中迁建项目，来宾盛宾中学建成办学，力争实现高中阶段毛入学率达到普及目标要求。完善职业教育和培训体系，深化产教融合、校企合作，统筹抓好县级中专改革。推进高等教育内涵式发展。深化医药卫生体制改革，进一步加快医药卫生系统信息化建设，以家庭医生签约服务、医联体和县域医共体为抓手，进一步完善分级诊疗制度；加强基层基础能力建设，增加优质医疗资源，改善基层办医条件；全面启动中医药壮瑶医药服务能力提升工程。做好人口和计划生育服务工作。进一步完善城乡文化设施及其服务体系，繁荣文艺创作，打造艺术精品。新增广播、电视综合覆盖人口约9万人。加快文化领域专项改革，推进传统媒体与新媒体融合发展，加强文化遗产保护，开展“扫黄打非”行动。广泛开展全民健身运动，推进 “全民健身和全民健康深度融合”试点建设，举办好第四届来宾市运动会等系列活动。

健全社会治理体系。坚守安全底线，深化平安来宾建设，严密防范和依法打击各类违法犯罪活动。继续打好禁毒人民战争。创新社会治理方式，打造共建共治共享社会治理新格局；推动公共安全信息化平台建设，深化综治中心建设，实现市县乡村四级全覆盖，筑牢社会治安立体化防控体系。加强源头治理，开展影响社会稳定的矛盾问题排查化解，进一步强化社会稳定风险评估工作，积极预防和化解“三大纠纷”等矛盾纠纷。完善公共法律服务平台建设，深入落实“谁执法谁普法”的普法责任制，推进法治来宾建设。进一步健全利益表达和协调机制，完善行政复议、劳动仲裁、阳光信访等渠道，切实保护群众的合法权益。加强重点行业、重点领域的安全监管，健全安全生产应急救援管理体系，深化安全隐患排查治理体系建设。继续强化食品药品监管工作。健全社会应急联动和预警监测机制，提高突发事件应对能力，确保社会和谐稳定。

（九）围绕政府建设，推动政务效能优化提升。

着力增强法治意识。严格依法履职，全面提高政府法治思维和依法行政能力，严格规范公正文明执法。全面落实市委的决策部署，自觉接受市人大及其常委会的法律监督、市政协的民主监督和社会的舆论监督，高质量办好人大议案建议和政协提案。坚持依法、民主、科学决策，建立重大决策预公开制度。完善政府法律顾问制度，支持检察机关依法开展公益诉讼工作。全面落实意识形态责任制。

着力增强服务意识。大力开展“高标准、高品质、高效能”主题创建活动，打造政务服务升级版。提升政务服务实体大厅服务功能，全面推行“一窗式”审批服务机制，实现办件受理、审管分离。推进“互联网+政务服务”深度融合，建设网上政务服务大厅，实现线上线下协同发展，让数据多跑路、群众少跑腿。继续深化行政审批制度改革，坚持推进“放管服”改革，切实转变政府职能。进一步理顺“三区”审批事项权责关系。深化推行市县乡三级权责清单制度。全面梳理和公开公共服务事项，最大程度方便群众和企业办事创业。开展好“不忘初心、牢记使命”主题教育。

着力增强清廉意识。打铁必须自身硬，全面从严治党要求我们必须全面从严治政、依法行政。坚持把政治纪律和政治规矩挺在前面，严格落实政府党组全面从严治党主体责任，以更高标准加强政府系统党风廉政建设。始终坚持无禁区、全覆盖、零容忍，保持反腐败高压态势，规范行政权力运行，习惯在受监督和约束的环境中工作生活。持续巩固拓展中央八项规定精神成果，强化扶贫领域监督执纪问责，持之以恒整治“四风”问题。全力支持国家监察体制改革试点工作。

着力增强担当意识。美好的蓝图，如果不落地，终究是水月镜花、一纸空谈。要健全奖勤罚懒的考核激励机制和容错纠错机制，严厉整肃庸政懒政怠政，坚决整治不作为、慢作为、乱作为，努力营造改革创新、干事创业的浓厚氛围。大力发扬苦干实干作风，提振勇于担当、主动作为的精气神。要以“心容天下 敢为人先”的决心和勇气，不怕困难、不怕矛盾、不怕辛苦，在加快发展中担当重任，干在实处，走在前列，狠抓各项决策部署落地见效，高质高效完成全年目标任务。

支持国防动员和后备力量建设，推进军民融合深度发展。保障妇女、儿童、老年人、残疾人合法权益。加强与工会、共青团、社科联、文联、红十字会等群团组织的联系。加强国家安全、防震减灾、机关事务、民族宗教、外事侨务、政策咨询、机构编制、统计、档案、气象、水文、人防、消防、保密、对台和地方志等工作。继续加强与金融保险、税务、石油、电力、烟草、通信、邮政、盐务、海事等部门的联系。凝聚社会共识，汇聚磅礴力量，共同创造美好生活！

各位代表!新时代要有新气象新作为，新征程要有新责任新担当。让我们紧密地团结在以习近平同志为核心的党中央周围，在自治区党委、政府和市委的坚强领导下，不忘初心，牢记使命，锐意进取，埋头苦干，奋力谱写新时代来宾发展的新篇章，以优异成绩迎接改革开放40周年和自治区成立60周年！
